# Contribution of Substance Use in Acute Injuries With Regards to the Intent, Nature and Context of Injury: A CHIRPP Database Study

**DOI:** 10.7759/cureus.10282

**Published:** 2020-09-06

**Authors:** Catherine Michaud-Germain, Pier-Alexandre Tardif, Alexandra Nadeau, Ann-Pier Gagnon, Éric Mercier

**Affiliations:** 1 Family and Emergency Medicine, Laval University, Quebec, CAN; 2 Axe Santé Des Populations Et Pratiques Optimales En Santé, Centre De Recherche Du Chu De Québec - Université Laval, Quebec, CAN; 3 Emergency Department, CHU De Québec - Université Laval, Quebec, CAN

**Keywords:** trauma, intentional injury, intoxication, self-harm, emergency medicine

## Abstract

Introduction

Using the Canadian Hospitals Injury Reporting and Prevention Program (CHIRPP) sentinel surveillance system, the objective of this study was to compare intent, circumstances, injury type and patient demographics in patients who used a substance prior to the injury versus those who did not use any substances.

Methods

Data were retrospectively collected from November 1^st^ 2016 to October 31^st^ 2017. All patients presenting to the Hôpital de l’Enfant-Jésus ED following trauma were included, aside from those who left without seeing a physician or had no physical injury (e.g., overdose without any trauma was excluded). Patients voluntarily completed a standardised form or agreed to be contacted later. Medical charts of all attendances were reviewed by the CHIRPP’s program coordinator. Substance use included illicit drugs, medications for recreational purposes, alcohol or other used either by the patient or another person involved.

Results

A total of 12,857 patients were included. Substance use was involved in 701 (5.5%) cases and was associated with injuries sustained by males (p < .001). The mean age of patients injured while using substances was 42.8 years, compared to 45.5 years in those who did not use substances (p < .001). Substance use was involved in 3.6% of unintentional injuries, compared to 26.2% of injuries intentionally inflicted by other and 38.9% for self-inflicted injuries (p < 0.0001). When substances were used, the odds of intentional injuries were 7.5 times greater compared to non-intentional injuries (95% CI 6.7, 8.5). Burns, head injuries and polytraumas were more prevalent when drugs or alcohol were involved.

Conclusion

This study outlines the significant contribution of substance use in intentional injuries, suggesting that it could potentially be beneficial to specifically target patients who present with deliberate physical injuries in preventive and therapeutic interventions offered in the ED.

## Introduction

Alcohol and drug use was found to be associated with traumatic injuries [1]. It was estimated that substance use disorder has caused 318,000 deaths globally in 2016 [2]. It has been shown that the odds of injury for those individuals consuming specifically alcohol were 5.0 times greater compared to non-exposure individuals in general population. Among drivers, those odds of injury were almost of 15 times greater [3]. Concerning illicit drugs, it has been shown that traumatic patients who screened positive were more likely to develop infectious complications [4]. Among teenagers, one study has shown that alcohol, marijuana and pain reliever use disorders are significantly associated with self and other-directed violence [5]. Another study has raised a red flag concerning a high rate of children (13 to 19 years) positive for alcohol or drugs following a trauma-related injury, especially among those with intentional mechanism of injury [6]. However, substance use and trauma is difficult to associate together as a wide array of terms is used to define a substance use and patterns of trauma [7]. Nevertheless, vigilance should be called for when facing a patient with a trauma-related injury to adequately treat or refer him. The best way to improve the quality of care of people who have been potentially injured under the influence of substances is to increase awareness of situations in which there is a suspicion that the injury occurred in a context of substance use.

While most studies that have explored the link between substance use and injuries in the emergency department (ED) among adults focused on the substances’ nature [8-10], data relative to the patients’ and injuries’ characteristics associated with a general substance use are lacking. To our knowledge, only one other study has examined simultaneously the characteristics of injuries caused during substance use and intent status of the injury in a socioeconomic country similar to Canada [11]. However, it also focused on substances’ nature.

Our objective was to compare intent, circumstances, injury type and patient demographics in patients who used a substance prior to the injury versus those who did not use any substances.

## Materials and methods

Data source

The Canadian Hospitals Injury Reporting and Prevention Program (CHIRPP) is a sentinel surveillance system conducted under the supervision of the Public Health Agency of Canada and designed to analyze events’ circumstances and identify risk factors that prevention strategies could target [12]. In this context, each injured patient presenting to a Canadian ED in which the CHIRPP program is implemented was asked to voluntarily complete a standardised form or to be contacted at a future date regarding this injury. More specifically, questions pertaining to understand how the injury occurred and what was the patient doing, the etiology of the injury, the factors contributing to the injury, the moment and the emplacement where the injury occurred, and finally the age and sex of the patient. In the case of intoxicated patients, invitation to fill the form was done only before discharge, when patients were sober again. All attendances were later reviewed by the CHIRPP’s program coordinator, who completed the unfilled or incomplete forms based on medical charts (including the nature of the injury, injured body part and treatment received).

Population and variables

This is a retrospective study in which data collection was undertaken at the Hôpital de l’Enfant-Jésus, a University affiliated hospital which is one of the three trauma centres in the Province of Quebec and a participating hospital into the CHIRPP program. All patients presenting to the Hôpital de l’Enfant-Jésus ED between November 1st 2016 and October 31st 2017 and seeking medical care for a traumatic injury or intoxication were included. Considering the focus on acute injuries, patients without any physical injury were thereafter excluded, along with those who left before seeing a physician since their information was unavailable. Moreover, because of potentially missing data, out-of-province/country patients were also excluded. Substance use was a categorical variable that indicated if illicit drugs, medications for recreational purposes, alcohol or other were used either by the patient or another person involved. It was considered as a factor in the injury event occurrence for values ‘yes’ and ‘suspected’. The intent of injury is dichotomized into the database between unintentional and intentional, the latest including self-harm, assaults and maltreatment. The injuries were categorized according to context, nature, body parts and intent as per the CHIRPP’s classification. The contexts of injury were grouped into eight different categories (see Appendix for details concerning items into each category).

Data analysis

Continuous and categorical variables were described using means (standard deviation) and proportions, respectively. The association between intoxication and categorical or continuous variables was evaluated using two-tailed Fisher’s exact test or Student’s t-test with Satterthwaite correction for unequal variances. The association between intoxication status and intent of injury was assessed using relative risks (RR) and 95% confidence intervals (CI) obtained with a modified Poisson regression model adjusted for age and sex [13]. Analyses were performed using Statistical Analysis System 9.4 (SAS Institute Inc., Cary, NC, USA). The level of statistical significance for all tests was set at p < 0.05.

## Results

During the study period, 16,275 patients visited the Hôpital de l’Enfant-Jésus ED following a trauma. Following the exclusion of patients out-of-province/country, those who left before seeing a physician and those without any physical injury, 12,857 patients were included. Of these, 56.8% (n = 7,302) were male and a great majority (n = 10,973, 85.3%) lived in urban areas.

Association with substance use

Substance use was involved in 701 (5.5%) cases and was associated with injuries sustained by males (p < .0001) (Table 1). The mean age of patients injured while using substances was 42.8 years, compared to 45.5 years in those who did not use substances (p < .001). Compared to patients with unintentional injuries, those reporting intentional injuries were significantly more likely to report substance use (39.4% vs 5.1%, p < 0.0001). The most favourable context for injuries in people who have used substance was during motor vehicle accident (11.7%), followed by low-risk activities (5.4%), while sports (17.5%) and work (15.7%) were the two most predominant contexts of injuries for those with no substance involved. Wound, head injuries and traumatic brain injury (TBI), and musculoskeletal injuries were the three most observed mechanisms in both groups. Proportions of burns, wounds, head injuries and TBI, and polytraumas were however significantly higher when substances were used (p < 0.02). Injuries primarily affected the head and neck when a substance was involved, while the spine and extremities were most implicated when no substance was involved. Supplemental material presents the patient characteristics stratified by intoxication status, but also classified by age categories.

**Table 1:** Characteristics of physically injured patients seen by an emergency physician stratified by intoxication status †More than three injuries; TBI: traumatic brain injury; *<0.05

		Substance used (n = 701)		Substance unused (n = 12,156)	P-value
Sociodemographic					
Age, mean (SD)		42.8 (18.0)		45.5 (24.1)	<0.0001*
Sex		-		-	<0.0001*
Female, n (%)		206 (29.4)		5,349 (44.0)	-
Male, n (%)		495 (70.6)		6,606 (56.0)	-
Geographic status		-		-	0.60
Urban, n (%)		603 (86.0)		10,370 (85.3)	
Intent of injury					
Unintentional, n		425 (60.6)		11,530 (94.9)	<0.0001*
Intentional, n		276 (39.4)		626 (5.1)	
Inflicted by others		127		358	-
Self-inflicted		79		125	-
Other or unknown		70		143	-
Injury					
Context, n (%)					
Work		3 (0.4)		1905 (15.7)	<0.0001*
Motor vehicle collision		82 (11.7)		1243 (10.2)	0.21
Other types of transport		18 (2.6)		390 (3.2)	0.35
Low-risk activities		38 (5.4)		916 (7.5)	0.04*
High-risk activities		0		134 (1.1)	0.01*
Sports		34 (4.9)		2132 (17.5)	<0.0001*
Aggressive attitude		29 (4.1)		68 (0.6)	<0.0001*
Information missing		0		60 (0.5)	0.06
Nature, n (%)					
Burn		22 (3.1)		229 (1.9)	0.02*
Electrocution		0		35 (0.3)	0.26
Foreign body		10 (1.4)		347 (2.8)	0.02*
Bite		6 (0.9)		133 (1.1)	0.71
Asphyxia		2 (0.3)		10 (0.1)	0.14
Wound		375 (53.5)		5125 (42.2)	<0.0001*
Traumatic amputation		1 (0.1)		64 (0.5)	0.27
Musculoskeletal		295 (42.1)		6139 (50.5)	<0.0001*
Head injuries and TBI		211 (30.1)		1408 (11.6)	<0.0001*
Polytrauma^†^		52 (7.4)		276 (2.3)	<0.0001*
Body part implicated, n (%)					
Head & neck		408 (58.2)		3477 (28.6)	<0.0001*
Spine		56 (8.0)		1277 (10.5)	0.03*
Trunk		82 (11.7)		1252 (10.3)	0.24
Extremities		241 (34.4)		7168 (59.0)	<0.0001*

Association with intend

Intentional injuries occurred in 902 (7.0%) cases, and were also associated with a lower average age (p < .0001) and male sex (p < .0001) (Table 2). A higher ratio of patients who were victims of assault lived in urban areas (p = 0.03). When substances were used, the odds of intentional injuries were 7.5 times greater compared to non-intentional injuries (95% CI 6.7, 8.5).

**Table 2:** Characteristics of physically injured patients seen by an emergency physician stratified by intent status †More than three injuries; TBI: traumatic brain injury; *<0.05

	Intentional (n = 902)	Unintentional (n = 11,955)	P-value
Sociodemographic			
Age, mean (SD)	33.6 (15.4)	46.2 (24.1)	<0.0001*
Sex	-	-	<0.0001*
Female, n (%)	298 (33.0)	5,257 (44.0)	-
Male, n (%)	604 (67.0)	6,698 (56.0)	-
Geographic status	-	-	0.003*
Urban, n (%)	800 (88.7)	10,173 (85.1)	-
Injury			
Context, n (%)			
Work	83 (9.2)	1825 (15.3)	<0.0001*
Motor vehicle collision	19 (0.2)	1306 (10.9)	<0.0001*
Other types of transport	1 (0.1)	407 (3.4)	<0.0001*
Low-risk activities	31 (3.4)	923 (7.7)	<0.0001*
High-risk activities	0	134 (1.1)	0.001*
Sports	34 (3.8)	2132 (17.8)	<0.0001*
Aggressive attitude	89 (9.9)	8 (0.07)	<0.0001*
Information missing	0	60 (0.5)	0.03*
Nature, n (%)			
Burn	17 (1.9)	234 (2.0)	0.88
Electrocution	0	35 (0.3)	0.17
Foreign body	35 (3.9)	322 (2.7)	0.04*
Bite	74 (8.2)	65 (0.5)	<0.0001*
Asphyxia	9 (1.0)	3 (0.03)	<0.0001*
Wound	537 (59.3)	4963 (41.5)	<0.0001*
Traumatic amputation	0	65 (0.5)	0.03*
Musculoskeletal	297 (32.9)	6137 (51.3)	<0.0001*
Head injuries and TBI	125 (13.9)	1494 (12.5)	0.23
Polytrauma^†^	36 (4.0)	292 (2.4)	0.004*
Body part implicated, n (%)			
Head & neck	402 (44.6)	3483 (29.1)	<0.0001*
Spine	33 (3.7)	1300 (10.9)	<0.0001*
Trunk	112 (12.4)	1222 (10.2)	0.04*
Extremities	400 (44.4)	7009 (58.6)	<0.0001*

Compared to patients with unintentional injuries, those reporting intentional injuries were significantly more likely to sustain their injuries via aggressive attitude (9.9% vs 0.07%, p < 0.0001) toward themselves or resulting from a fight with a third party. The second context in frequency among intentional injuries is during work. Proportions of foreign body injuries, bite, asphyxia, wounds and polytraumas were significantly more frequently sustained intentionally (p < 0.04).

## Discussion

This is the first study using the CHIRPP database that portrays the contribution of substance use, regardless of the substance’s nature, and the intend surrounding the acute injuries.

Key findings of this study highlight the substantial contribution of substance use to intentional injuries seen in the ED (39.4% vs 5.1%, p < 0.0001). This concurs with a random sample of ED patients in Vancouver (British Columbia, Canada) in which alcohol and recreational drug use, alone or in combination, were both strongly associated with intentional injuries [11]. As opposed to other studies relying on patients’ urine or blood analysis, this study allowed for these bilateral associations to be brought into light by considering that substance use was also a contributing factor when someone other than the patient was intoxicated and involved in the injury. Another interesting discovery is that people who harm themselves will do so in a fit of anger, almost as much as when they are working.

The ED provides a unique opportunity for screening and early intervention for behaviours such as alcohol and substance use [10]. It is often the interface between the traditional health care system and vulnerable populations such as homeless persons [14], domestic violence victims [15] and older adults who are known to be at risk of violence-related events and substance abuse [16]. Various studies support the implementation of substance use screening tools for trauma patients in the ED [1, 10] and our results suggest that patients with deliberate physical injuries could potentially deserve greater screening.

The results also stress the importance of making intervention procedures available for the intentionally injured population. Unfortunately, some physicians still perceive brief interventions for substance use as ineffective despite how they were shown to decrease consumption, arrests, recidivism and injuries [1]. For instance, ED-initiated interventions to reduce subsequent suicidal behavior in high-risk patients [17] as well as ED-based programs aiming to reduce alcohol-related violence have been successfully implemented [18]. Alcohol intoxication has been linked to trauma recurrence [19]. Furthermore, patients with self-harm injuries are at known high risk of recidivism [20, 21]. In that context, individualized interventions would be beneficial, but those types of ED-based interventions are infrequently available.

Limitations

Using patients from the CHIRPP program allowed a larger sample size. However, the conclusions reached should be cautiously generalized since the sample was not population-based and some subgroups might be underrepresented due to the hospital’s status and location. In addition, the associations found cannot be interpreted in terms of causation, notably due to study design being retrospective and modelling (possible residual confounding). Nevertheless, systematic and standardized data collection allowed to representatively picture consultations for acute injuries in the ED of a tertiary trauma centre in Quebec, Canada. Considering intentional injuries, we do not have the information as to whether it was the victim, the assailant, or even both individuals who were intoxicated. In addition, the few cases of assaults or self-harm did not permit to create two distinct groups for comparison regarding the intent. Details on injuries and poisoning circumstances were not specifically studied, for which the use of verbatim would have added other limitations. With that said, this is of minor importance considering the aim of the study was not to describe the mechanism of injuries but rather to determine the types of injuries in which substance use played a significant role. Moreover, a misclassification bias is probable since solely CHIRPP variables were used. However, this bias is likely negligible since a recent report suggests CHIRPP variables have very high sensitivity; data mining with narrative codes would not have improved the classification’s accuracy [22]. Finally, as data were self-reported or extracted from the medical record, an underestimation relative to the contribution of substance use is possible. Further details regarding the psychological [23] and homelessness [24] history of patients, as well as their financial status [25], would have potentially allowed additional conclusions regarding substance use and intend in acute injury.

## Conclusions

This study, using the CHIRPP sentinel surveillance system, outlines the fact that a significant proportion of the population were injured because they were using substances, especially those whose injuries were intentional or the results of aggression. It could potentially be beneficial to target patients with deliberate physical injuries in preventive and therapeutic interventions offered in the ED. It stands to reason that encouraging the use of screening tools, biopsychosocial assessment (including evaluation of substance use) and brief interventions in this population, as well as ensuring adequate training of ED staff for this purpose, should also be considered as strategies to limit the considerable impact of substance use on intentional injuries.

## Figures and Tables

**Figure 1:**
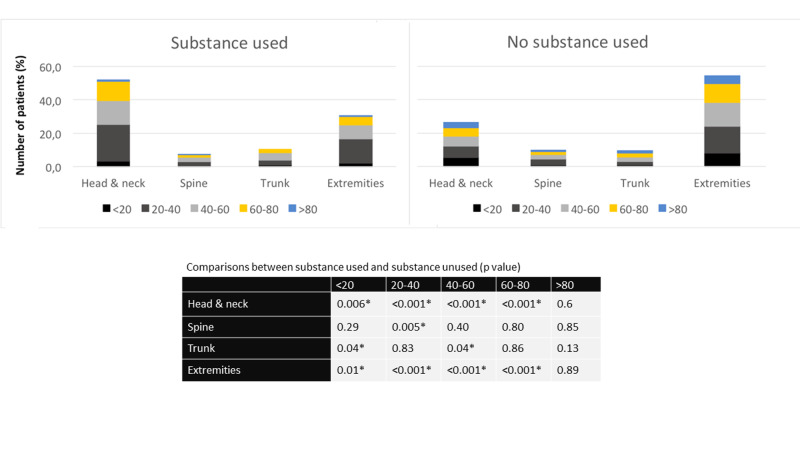
Body parts injured by intoxication status and by age category * p < 0.05

**Figure 2:**
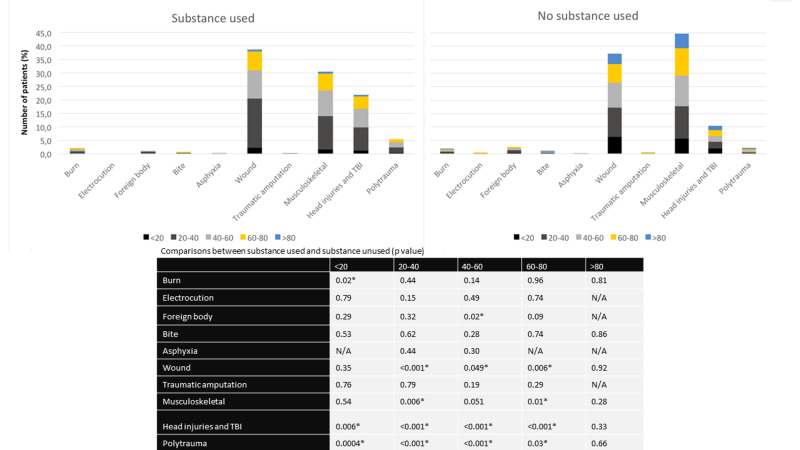
Nature of injury by intoxication status and by age category TBI: Traumatic brain injury; * p < 0.05

**Figure 3:**
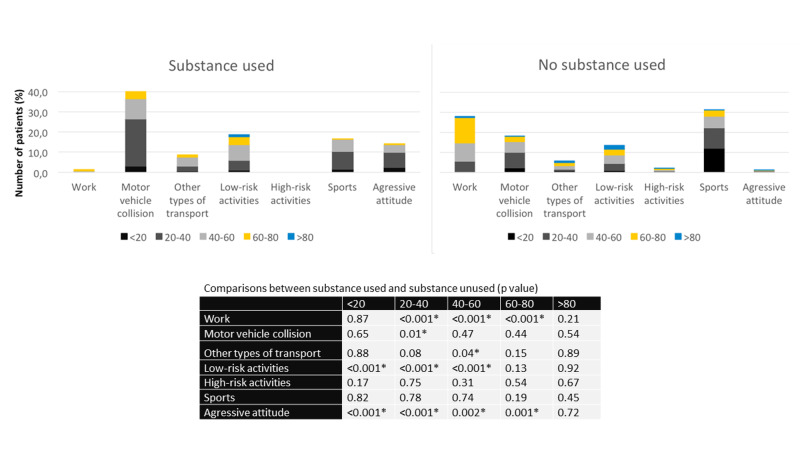
Context of injury by intoxication status and by age category * p < 0.05

## Appendices

**Table 3:** Grouping of contexts of injury

Work	N/A
Motor vehicle collision	Riding on motorcycle/scooter (2 wheels), including driver, passenger, motocross, dirt bike
	All-terrain vehicle
	Snowmobile, being towed by snowmobile
	In car or passenger van, jeep
	In other transport, including train, plane, tractor
	Using wheelchair/powered or unpowered, mobility assistance devices
	All watercraft, including powered and unpowered, private and commercial, sail boat
Other types of transport	Pedestrian, including baby being carried or child in stroller on street
	Bicyclist
	Walking, running, crawling
Low-risk activities	Recreation or hobby activities, including model building, brownies, crafts, partying, music lessons, scouts, watching TV, drinking with friends
	Laundry
	Food preparation (no heat)
	Cooking, including making tea, coffee, hot beverage
	Cleaning, including dish washing
	Eating or drinking
	Washing, showering, bathing
	Sleeping, resting
	Dressing
	Sexual activity
	Other personal activity, including hairdresser, tattoo, beautician, physio rehabilitation
	Sitting, standing - passive, carrying a child
	Shopping
	Being cared for, including patient in hospital, MD's office; person being dressed, bathed, fed
	Playing, including climbing inside at home
	Other activity inside/outside, including bringing in groceries, taking out garbage, loading/unloading vehicle, household chores, lifting vacuum cleaner, getting down from a chair, cutting metal
	Formal educational activities, including homework, teaching/coaching sports
	Volunteer (unpaid work outside home), including fundraising
High-risk activities	Electrical work
	Do-it-yourself, including plumbing, carpentry, etc.
	Vehicle maintenance
	Moving furniture
	Gardening, yard work, including chopping wood, cutting trees, shoveling snow, taking in wood
Sports	Sports, organized competition or practice and physical recreation activities, including lessons, physical education
	Sports, informal and physical recreation activities including tobogganing, trampolining, dancing (in home), hide-and-seek, tag, playfighting
	Sports, not specified
Aggressive attitude	Had tantrum, loss of temper, loss of control (only one person involved)
	Quarrel, aggression, fight, riot (two or more people involved)
	Intent to cause effect or reaction (intent to cause effect to oneself or reaction by another), including acting out, self-harm
Information missing	N/A
